# Integrative approaches for analysis of mRNA and microRNA high-throughput data

**DOI:** 10.1016/j.csbj.2021.01.029

**Published:** 2021-01-26

**Authors:** Petr V. Nazarov, Stephanie Kreis

**Affiliations:** a Multiomics Data Science Research Group, Department of Oncology & Quantitative Biology Unit, Luxembourg Institute of Health (LIH), Strassen L-1445, Luxembourg; b Signal Transduction Group, Department of Life Sciences and Medicine, University of Luxembourg, Belvaux L-4367, Luxembourg

**Keywords:** CCA, canonical correlation analysis, CDS, coding sequence, circRNA, circular RNA, CLASH, cross-linking, ligation and sequencing of hybrids, CLIP, cross-linking immunoprecipitation, CNN, convolutional neural network, GO, gene ontology, ICA, independent component analysis, lncRNA, long non-coding RNA, miRNA, microRNA, mRNA, messenger RNA, NGS, next-generation sequencing, NMF, non-negative matrix factorization, PCA, principal component analysis, RNASeq, high-throughput RNA sequencing, TDMD, target RNA-directed miRNA degradation, TF, transcription factors, microRNA, Transcriptomics, Data integration, Target prediction, Matrix factorization

## Abstract

Advanced sequencing technologies such as RNASeq provide the means for production of massive amounts of data, including transcriptome-wide expression levels of coding RNAs (mRNAs) and non-coding RNAs such as miRNAs, lncRNAs, piRNAs and many other RNA species. *In silico* analysis of datasets, representing only one RNA species is well established and a variety of tools and pipelines are available. However, attaining a more systematic view of how different players come together to regulate the expression of a gene or a group of genes requires a more intricate approach to data analysis. To fully understand complex transcriptional networks, datasets representing different RNA species need to be integrated. In this review, we will focus on miRNAs as key post-transcriptional regulators summarizing current computational approaches for miRNA:target gene prediction as well as new data-driven methods to tackle the problem of comprehensively and accurately dissecting miRNome-targetome interactions.

## Highlights

• Review on tools and databases linking miRNA and its mRNA targetome.
• Databases show little overlap in miRNA targetome predictions suggesting strong contextual effects.
• Deconvolution and deep learning approaches are promising new approaches to improve miRNA targetome predictions.

## 1 Introduction

An accurately fine-tuned regulation of the expression levels of the ~20 000 protein-coding genes and the far more abundant non-coding RNAs is essential for a healthy functioning of human cells. Gene regulation is achieved at different levels. Epigenetic mechanisms influence, prior to actual gene transcription, which gene is being transcribed in a given cell at a given time. Next, a multitude of intrinsic or extrinsic signals determine which transcription factors are activated downstream of signalling cascades to drive or inhibit transcription of target genes. At the post-transcriptional level, the half-life, stability and other factors affect the available amount of bioactive RNAs, but the most important cellular instrument to adjust expression levels of most classes of RNAs, are small non-coding RNAs called miRNAs (microRNAs).

A recent bioinformatic analysis of some 360 billion sequencing reads, revealed 2300 true human mature miRNAs, roughly half of which are annotated in miRBase V22 [1]. In humans, ~520 high-confidence miRNA canonical genes and ~120 conserved miRNA families (with similar seed sequences) have been identified [2] with each family targeting > 400 conserved mRNAs, altogether resulting in ~60% of all mRNAs being targeted by miRNAs [3].

miRNAs are remarkably stable 22 nt short oligonucleotides that are produced from miRNA-encoding genes in a well understood biogenesis process comprehensively reviewed elsewhere [2]. Mature miRNAs act as guide sequences directing the RNA-induced silencing complex (RISC) to target RNAs, which contain complementary binding sites that allow for a miRNA:target contact. The canonical and biologically most relevant interaction takes place between the seed region (5′ nucleotides 2–7) of a miRNA and the binding site in the 3′ UTR of an mRNA target, resulting in a rapid degradation of the mRNA or less frequently in an inhibition of its translation into protein [4]. It has been estimated that 100 s-1000 s of miRNA molecules are necessary to efficiently repress mRNA levels in a cell [5].

Although canonical interactions are functionally most relevant, the highly abundant non-canonical binding to regions outside the 3′UTR of mRNAs and involving nucleotides beyond the seed sequence within the miRNA as well as miRNA binding to other non-coding RNAs are likely contributing to post-transcriptional gene regulation by competing with canonical binding events and by occasionally leading to down-regulation of the non-canonical target itself [6], [7], [8] and own unpublished data [9]. Other non-coding RNAs such as long non-coding RNAs (lncRNAs), competing endogenous RNAs (ceRNAs) and circular RNAs (circRNAs) have also been shown to sequester miRNAs from the biologically active pool within the cell [10], [11]. Further contributing to the complexity of post-transcriptional gene regulatory networks is the fact that the expression of miRNAs themselves is also a dynamically regulated process, involving above mentioned pre-transcriptional epigenetic and genetic mechanisms, transcription factors and signaling pathways, which are generally cell type- and disease-specific and have relevance for most physiological processes as well as in complex diseases such as cancer [12], [13]. Finally, there is redundancy in miRNA-target gene interactions and different related or unrelated miRNAs might have to cooperate to induce a measurable effect on gene expression. Likewise, for an observable change in cellular phenotypes, expression changes in only one gene are often not sufficient and several genes might have to be regulated at the same time for a functional shift in cellular behavior.

Although target gene prediction has markedly improved over the last decade, we are still far from being able to predict the miRNA targetome with an acceptable quota of false positive and false negative results, although many tools are available for prediction of miRNA target genes [14], [15], [16]. The aforementioned properties and the problematic target gene prediction make miRNA-driven gene expression difficult to unravel in all its complexity. Only massive amounts of well-controlled, high quality and preferably dynamic data (collected at various time points) from different cells/tissues from healthy and diseased conditions representing different transcriptional states can improve our understanding of the intricate regulatory circuits involving miRNAs. This being a costly and tedious endeavor might in part explain why the initial enthusiasm on miRNA research some 10–15 years ago has somewhat cooled down. It soon became clear that simply generating mono-phasic miRNA profiling data would not lead to the desired overview of transcriptional regulatory networks.

In the current review, we will provide a brief update on available computational miRNA tools and then focus on approaches of data integration efforts aiming at jointly analysing gene expression datasets representing miRNAs and mRNAs, lncRNAs or other non-coding RNAs. Advancing such integrative *in silico* analyses becomes even more important in view of an ever-growing number of publically available transcriptomic datasets.

## 2 Methodologies for analysis of miRNA-target gene interactions

### 2.1 Approaches to miRNA target identification

From the first identification of miRNAs in the 1990 s (lin-4 and let-7 in *C.elegans*, [17], [18]), tremendous efforts have gone into computational prediction, experimental detection and validation of miRNA target genes. Current experimental methods to analyse the miRNA targetome include: i) profiling expression levels of the entire transcriptome (by RNASeq or microarray) following overexpression or downregulation of a miRNA of interest; ii) measuring selected mRNA levels of predicted targets by qPCR and/or measuring levels of the corresponding proteins following overexpression or downregulation of a miRNA of interest; iii) crosslinking followed by immunoprecipitation of RISC complexes (CLIP and CLASH methods); iv) reporter gene assays with the target site of the miRNA cloned close to a reporter gene and with external delivery of the miRNA v) as well as profiling phenotypic traits following rescue of mutated or deleted miRNAs [4], [14], [19], [20], [21], [22].

Experimental methods can provide direct links between miRNAs and their targets, but they are not error-free, extremely laborious, time consuming and expensive, especially when more than one miRNA is investigated. Some experimental methods were criticized for generating false positive results [21] and this is even more so when analysing data from RNASeq experiments following overexpression of miRNAs. The recent introduction of CLASH [23] provides an interesting addition to the experimental tool box by directly linking miRNAs with the bound mRNAs, lncRNAs or any other potential RNA target. However, also this technology has room for improvement as the identification of miRNA-target hybrids is still rather inefficient [24].

Computational prediction of canonical or non-canonical miRNA targets employ different statistical and machine-learning approaches and generally analyse some of the following criteria: i) degree of Watson-Crick pairing between the miRNA seed region and target site; ii) evolutionary conservation across species; iii) thermodynamic properties; iv) accessibility of target sites; v) sequence composition in the vicinity of seeds and target sites. Many studies and comprehensive reviews have described available tools before [4], [8], [15], [25], [26], [27], [28] some of which will be further discussed below.

**Fig. 1:**
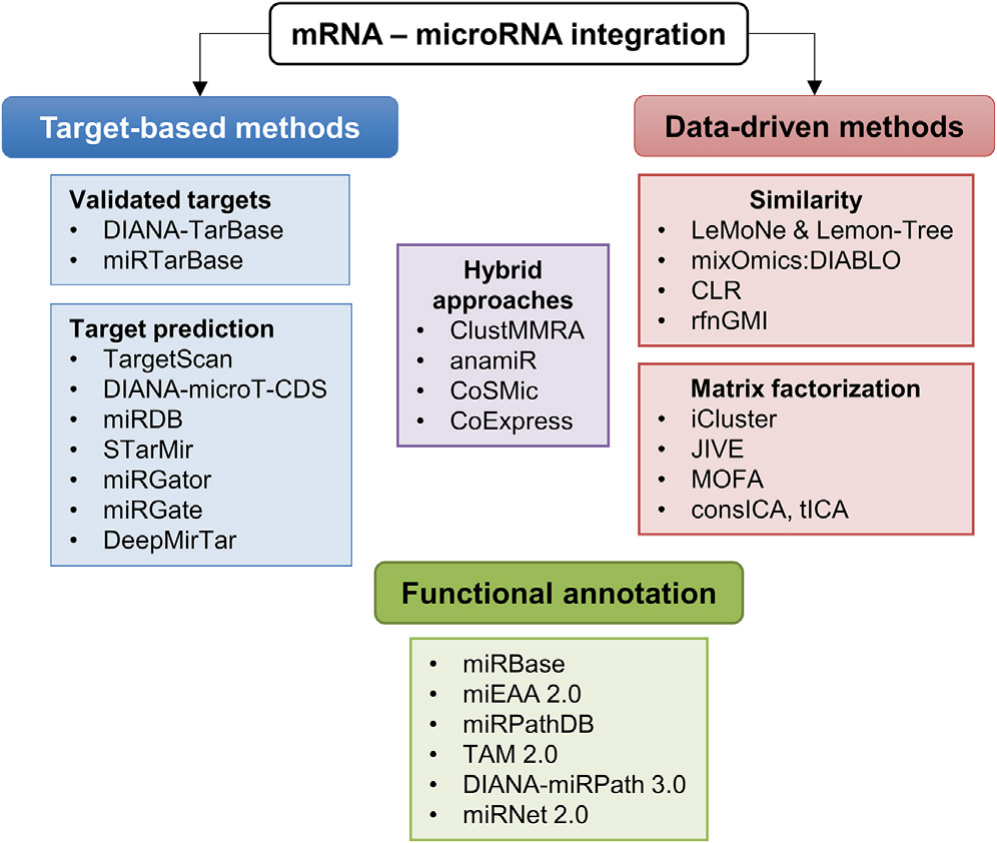
An overview of the main methods for miRNA:mRNA data integration.

With the development of high-throughput technologies in experimental genomics, it became feasible to detect complete transcriptomes and miRNomes of cells and tissues under various conditions. Expression profiles of matching miRNomes and transcriptomes from given cells or tissues and CLIP-based next generation sequencing (NGS) provide large datasets, in which the true interaction partners need to be identified and this is not a trivial task. Integration of different datasets generally explores statistical similarities and inverse correlations of mRNA and miRNA expression patterns, which can be suggestive of potential interactions or the presence of co-regulated clusters. Such knowledge would certainly contribute to our understanding of miRNA functions if the number of false positive and negative predictions can be reduced. Below and in Fig. 1, some of the most popular and promising methods for integration of mRNA and miRNA datasets are summarized.

### 2.2 Databases of experimentally validated miRNA targets

Over the past 10 years, several dozen online resources with predicted or validated miRNA targets have been published [25], [26]. However, many of them had quite a short life cycle and are currently either unavailable or outdated. Here we concentrate on databases that are regularly updated, starting with experimentally validated targets, as they are the most valuable source for miRNA-target gene pairs.

DIANA-TarBase is the most complete collection of experimentally supported miRNA targets. The current version 8 of the database contains around 670 k of unique miRNA-mRNA pairs (and over 1 M of entries of which ~800 k have direct experimental support). It has been persistently developed over the past decade and is based on both literature curation (1.2 k) and analysis of results of low- and high-throughput experiments [29]. Importantly, the database can be downloaded, which makes it an interesting source for an automatic computational analysis.

MiRTarBase is the second largest collection of experimentally validated targets [30]. The current version describes over 430 k miRNA-target interactions. It is based on manual curation of around 11 k publications and is also downloadable.

### 2.3 Tools for prediction of miRNA targets

TargetScan. Among the target gene prediction tools, TargetScan [28] is the most widely used. The tool and corresponding database have been supported and developed since 2005, with the current version 7.2 available since 2018, covering miRNA interactions in 8 mammals (including human, mouse and rat and several other organisms). Predictions by TargetScan are made by searching for conserved 6–8-mer binding sites in mRNAs, matching with miRNA seed regions and taking into account the surrounding sequences (in total, 14 distinct parameters were used for prediction). Prediction databases can be downloaded separately for conserved miRNAs with conserved mRNA targets and for non-conserved miRNAs with conserved and non-conserved mRNA targets (the most complete). Agarwal and colleagues showed that non-canonical sites rarely lead to mRNA modulation despite binding of miRNA, and therefore focused their predictions on the canonical sites [28]. However, given the increasing evidence that non-canonical interactions between miRNAs and their targets also play an important role in some (but not all) gene regulatory networks ([2], [6] and unpublished data in [9]), such interactions involving nucleotides outside the seed of the miRNA and within the CDS (rather than the 3′ UTR) represent useful additions to current prediction tools.

DIANA-microT-CDS. DIANA tools, in parallel to its validated target database introduced above also proposes the target-prediction by DIANA-microT-CDS, the 5th version of the microT algorithm. This method uses a machine learning approach that is trained on photoactivatable-ribonucleoside-enhanced cross-linking immunoprecipitation (PAR-CLIP) data and is able to predict miRNA binding sites both in 3′-UTR and in coding sequence (CDS) regions. The tool does not allow the download of predicted targets for all miRNAs, however it allows access to predictions through the Taverna workflow management system.

miRDB is an online resource for miRNA target prediction and functional annotations [31]. The method uses the machine-learning algorithm MirTarget based on a support vector machine, which takes evidences from several other algorithms, including TargetScan [28], PicTar [32], miRanda [33] and combines them with features obtained from miRNA overexpression experiments and CLIP data. The method allows for predicting gene targets with binding sites both in evolutionary conserved and non-conserved regions of 3′-UTR. Current lists of predicted targets are available for human, mouse, rat dog and chicken and can be downloaded from the miRDB server. In total, the database contains 3.5 M miRNA-mRNA target pairs and over 1.6 M human entries.

There are more tools available that build their predictions on combination of the above mentioned databases and algorithms such as the recently updated miRWalk [34]) or including experimental data, miRGator [35], STarMir [36] or miRGate [37]), but these tools seem to be applied less frequently for the moment. Another important property of such databases is the frequency of update. After a 5-year stand-by period, databases lose their attractiveness for the community and deviate too much from the current version of miRBase [38].

Recently, a deep learning approach, which is based on advanced artificial neural networks, has been applied to different tasks in bioinformatics [39]. Although neural networks have been known for decades as universal modelling tools (e.g. [40]) only now have computer power and, more importantly, the size of datasets reached the dimensions where efficient and robust networks can be built. A convolutional neural network (CNN), one application of deep learning models, has been successfully applied to predict binding affinities between miRNAs and 12-mer sequences [8]. The model substantially improved prediction of mRNA repression in cell lines. Considering these promising developments, we should expect to see more works dedicated to application of deep learning in miRNA target predictions. Another example of a deep learning approach is provided by the DeepMirTar tool [41] where authors used stacked de-noising auto-encoders to predict miRNA targets at the site level. Interestingly, the tool showed improved performance in target prediction compared to standard methods, including TargetScan.

### 2.4 Functional annotation

Functional annotation of individual miRNAs or related groups or families of miRNAs is important for understanding their biological roles and can also be useful to connect miRNAs to known functional gene sets. For a small number of selected miRNAs, their functions can be determined experimentally. However, high-throughput datasets require bioinformatics analysis with literature curation [42], [43] or functional analysis of miRNA targets [44]. The fact that miRNA and regulated mRNAs are linked by a “many-to-many” relationship, significantly increases the complexity of functional miRNA annotation. The most important tools are introduced below.

miRBase – the primary public database for miRNA sequences and nomenclature [38]. The current release 22.1 contains 38,589 entries for 271 organisms. Each entry represents a miRNA precursor sequence with a predicted hairpin of the miRNA transcript, the genomic location, references from literature, the mature miRNA with manually curated gene ontology (GO) terms [42] and other information. Since the first presentation in 2002, miRBase has been widely used as a reference catalogue by other miRNA-related tools. A somewhat adverse effect of miRBase popularity is a requirement of a high robustness for the presented information and, therefore, the lack of pilot tools incorporated. For example, while a single mature miRNA can be linked to GO terms, there is no enrichment analysis and functional annotation for a set or group of miRNAs. Therefore, other tools have to be used in order to obtain such annotations.

One such tool is the 2nd version of the miRNA Enrichment Analysis and Annotation package (miEAA 2.0), which aims at functional analysis of sets of miRNAs [45]. MiEAA is based on GeneTrail, an enrichment analysis tool for gene sets [46] and integrates data from different sources including miRBase, miRWalk, miRTarBase and others. MiEAA can work with lists of precursors and mature miRNAs and performs either over-representation analysis (Fisher exact test) or enrichment analysis (Kolmogorov-Smirnov test). Available categories include for example GO, KEGG pathways, target genes, chromosomal location, diseases, drugs (altogether 130 k categories). In parallel, the same group proposed another tool, miRPathDB. This dictionary, based on over-representation analysis of miRNA targets provides miRNA-centric, gene-centric and pathways-centric views [44].

**Fig. 2:**
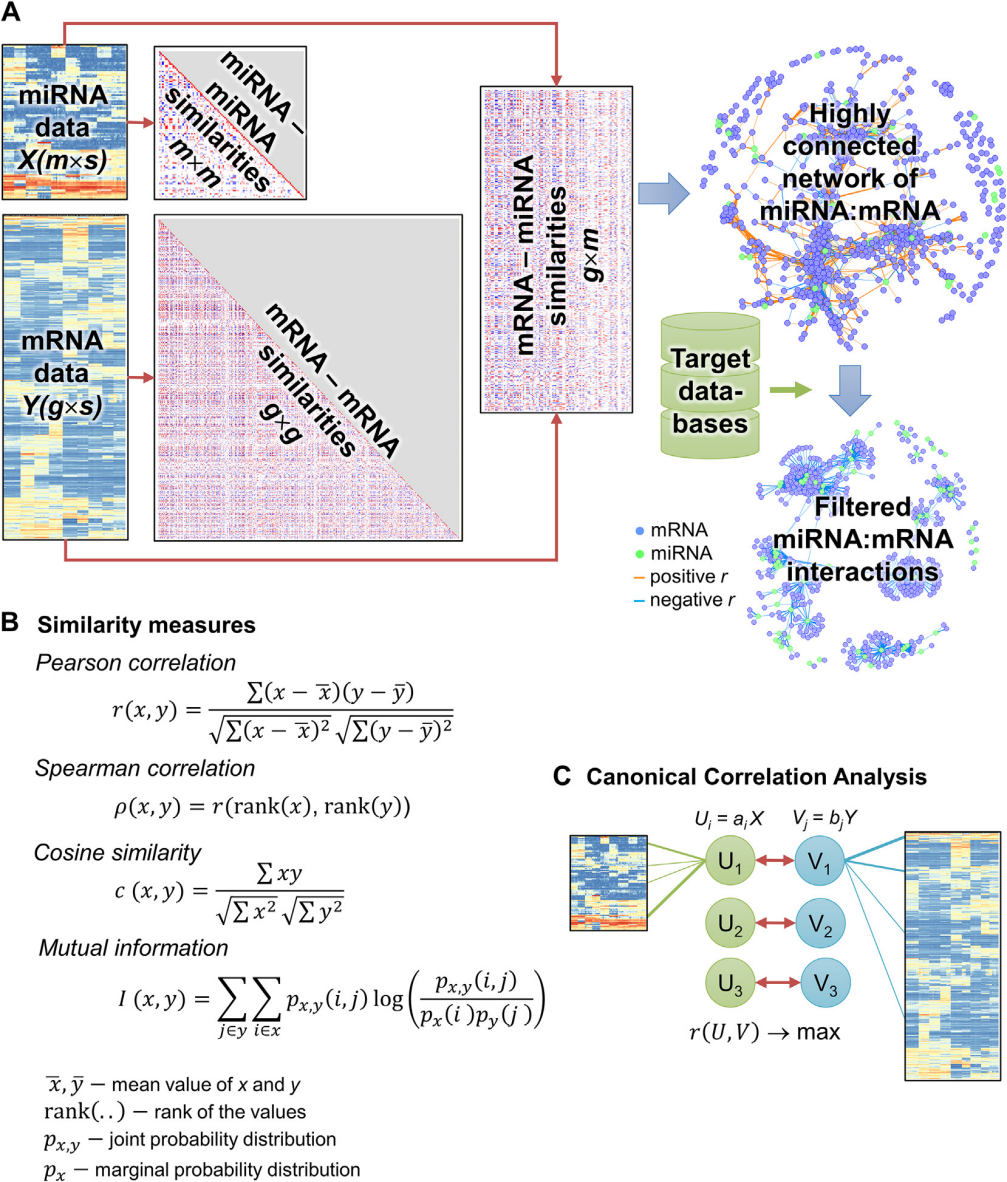
Similarity-based methods. (A) Correlation or other similarity measures produce a highly-connected network of miRNAs and potentially regulated mRNAs. Due to the high level of correlation between genes, such networks are often redundant and contain many false positives and should be filtered using miRNA target databases. (B) Standard similarity measures used for comparison of miRNA and mRNA profiles. (C) Canonical correlation analysis (CCA) approach: new features *U* and *V* are built as linear combinations of miRNA and mRNA profiles in a way that maximizes correlation between them.

Another tool, often used for functional annotation of miRNA is TAM 2.0 [43]. The tool is based on a manual curation of 9 k papers. It includes 1238 miRNA sets associated with different diseases, miRNA families, transcription factors and biological functions. With input lists of up- and down-regulated miRNA, TAM 2.0 can analyse deregulated miRNAs in two conditions by cosine similarity. This measure is based on the inner product of two vectors, and is sensitive to the means of these vectors. For centred vectors (zero mean), cosine similarity is equal to the Pearson correlation (see Fig. 2B). Importantly, in both miEAA and TAM, users can provide their own reference (background) set of miRNAs, reducing the bias.

DIANA tools also provide functional annotations – DIANA-miRPath 3.0 [47] works with both single and multiple miRNAs. Categories are represented by KEGG molecular pathways and GO in several organisms. The tool links miRNAs with regulated mRNAs using several target prediction algorithms (DIANA-microT-CDS, TargetScan) and validated targets (DIANA-miRTarBase). An implemented reverse-search module allows identifying miRNAs that control specific pathways. A drawback of the tool is the fact that reference miRNA or mRNA lists cannot be selected or changed.

A visual analytics and integration tool, miRNet 2.0 [48], provides users with the possibility to build networks based on own lists of miRNAs, mRNAs, transcription factors (TF) and other regulatory elements (12 modules in total). In cases where miRNAs are selected as a starting module, the tool builds a network of miRNAs and their targets (based on one of above mentioned databases) and visualizes it. It also allows for functional annotation based on the target list.

### 2.5 Data-driven methods: similarity-based

Accessibility of large transcriptomic datasets that represent mRNA and miRNA profiles across many samples and conditions facilitate the use of data-driven, hypothesis-free approaches to data integration. The simplest way to integrate different datasets would be the calculation of correlation within and between miRNA and mRNA profiles, which is possible if both data types were collected from the same samples or at least the same conditions (Fig. 2, the heatmaps and networks illustrate the basic idea but are based on real data from [49]). This approach, however, is unable to discriminate between direct and fake miRNA:mRNA interactions, originating from common hidden regulators such as transcription factors. Therefore, additional filters should be used to prioritize meaningful interactions. Such filtering can be done, for example, by considering only negatively correlated miRNA:mRNA pairs, where the mRNA is also predicted as a potential target of the miRNA [13]. Additional layers of complexity were brought in by the fact, that both mRNA and miRNA datasets have a high intrinsic correlation. This could originate from simultaneous activation or repression of genes and miRNAs participating in the same or linked functions. In order to deal with such behaviour, a module-based method, the Learning Module Network LeMoNe was proposed and applied to infer functions and regulated targets of miRNAs [50]. Their two-step algorithm includes a partition of genes into co-expressed clusters followed by inferring a regulatory program for each cluster. This method was further developed in a cross-platform open source Lemon-Tree tool [51].

Another approach is built on canonical correlation analysis (CCA), a classical method to establish linear relations between two sets of correlated observations. The method accepts the fact that experimental data are correlated and builds linear combinations of features for both datasets in a way to maximize correlation between them. One of the most successful example of this approach is DIABLO, an integrative method based on sparse generalized CCA [52]. This method can be used to identify markers and to build links between multi-omics data and patient groups. It was specifically tested on integration of miRNA and mRNA datasets. The method is implemented as a part of a powerful mixOmics R-package, able to integrate results across several omics datasets or derived from different studies [53]. Recently, another useful application of CCA was applied to identify miRNA-disease associations [54].

Pearson correlation captures only linear dependency between expression of mRNA and miRNA. Changing to Spearman rank correlation could broaden this to a wider range of monotonic dependencies. However, in reality miRNA:mRNA dependency can be more complex, and requires considering two-dimensional distributions between miRNA and mRNA expression. In [55], triangular-shaped patterns between miRNA and its targets were reported. The authors proposed an “antagonism pattern detection algorithm”, based on counting and statistical assessment of observations in lower and upper triangles of a scatter plot. Alternatively, mutual information can be used to detect non-random profiles. Mutual information is able to capture different non-linear patterns and is used in the context likelihood of relatedness (CLR) algorithm [56]. As an example of a successful application, this method allowed for identification of miRNA regulatory network in glioblastoma [57].

Several authors addressed the problem of context- or condition-specific relations between miRNAs and their targets [58], [59], [60]. Context-specificity can be taken into account using biclustering, a method to cluster subsets of genes that have similar expression in a subset of conditions [58], [59]. For instance, the rectified factor network-based biclustering for genes, miRNAs and interactions (rfnGMI), presented by Su et al. [59], was applied to detect genes and miRNA markers specific to breast cancer.

### 2.6 Data-driven methods: Matrix factorization

**Fig. 3:**
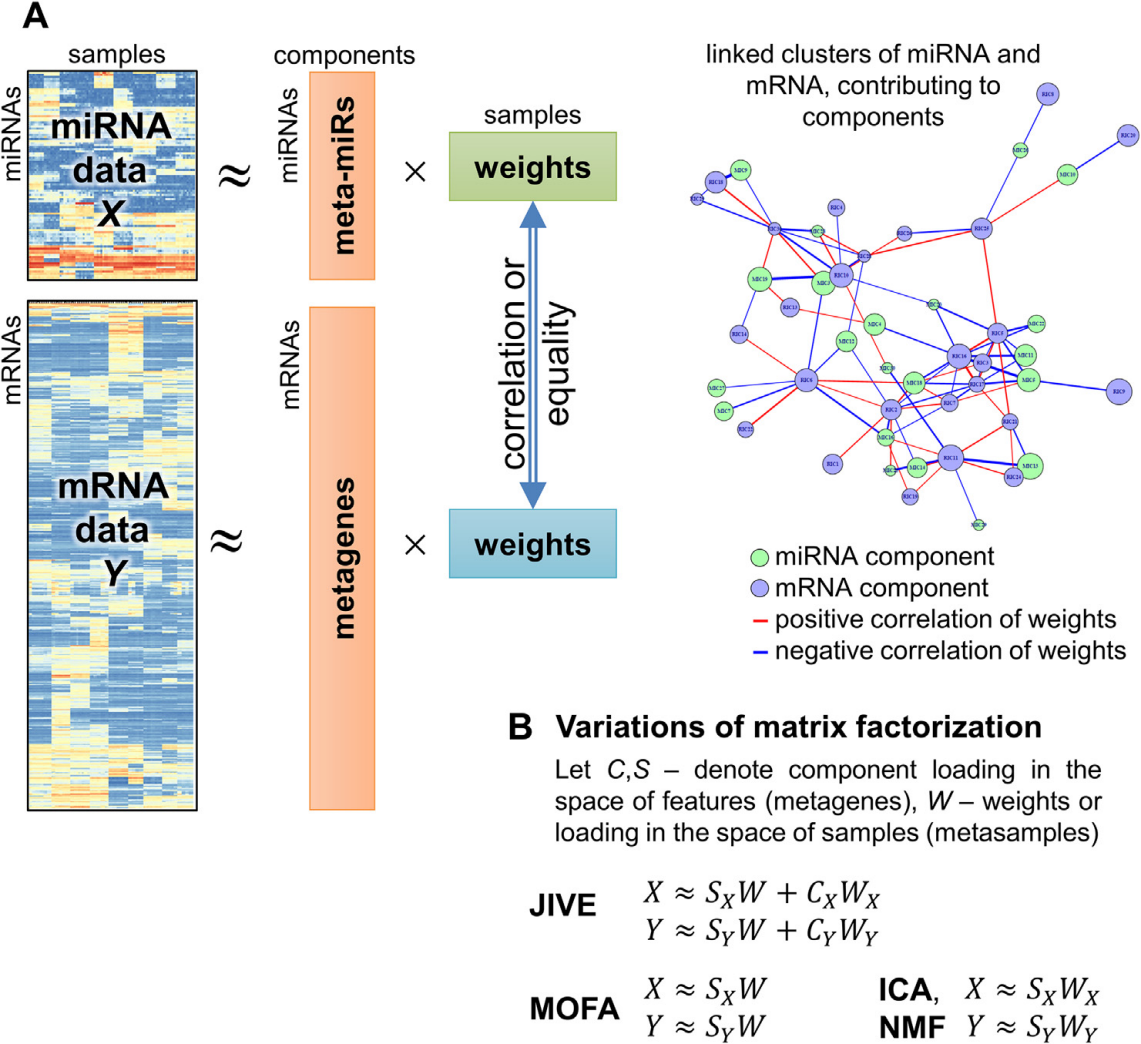
Matrix factorization methods. (A) Each expression matrix is presented as a product of two lower-rank matrices. Integration can be performed by correlating weight profiles over the samples resulting in a network of linked components. Some methods (e.g. MOFA) use a single weight matrix for both datasets. (B) Variation of the approach in different methods: JIVE, MOFA and ICA. The classical NMF approach has the same mathematical expression as ICA, but requires that both metagene (*S*) and weight (*W*) matrices are composed of non-negative elements.

Matrix factorization methods originally represent the expression matrix as a matrix product of lower rank matrices with the original mRNA and miRNA data measured in *m* samples (Fig. 3). We assume that there are some hidden (latent) variables or components that are shared between data types. These variables can for example, explain variability of the data over subgroups of samples (cancer subtypes or patient outcomes). The effect of a latent variable on features (genes, miRNAs) is described by the “metagene” matrix, the effect on samples by the “weight” matrix. Moreover, the weights of the same latent variables affecting mRNA and miRNA data should either be the same, or should at least be correlated. By linking these variables, it is possible to integrate the data, linking miRNAs and mRNA belonging to the same latent variable.

Various methods of building this matrix product and properties of the resulting matrices have been proposed. Here we describe several of the most widespread approaches. Some of them focus on general integration of multi-modal (multi-omics) data, which can also be projected onto miRNA and mRNA data integration. The first attempt to develop matrix factorization for linking sets of mRNA and miRNA was performed within the SNMNMF tool (Sparse Network-Regularized Multiple Non-negative Matrix Factorization) [61], which is, unfortunately, not available anymore. The tool employed non-negative matrix factorization (NMF) [62], which requires matrix elements to be non-negative. Although the method fits well to the physical nature of non-negative RNA quantities, it suffers from ambiguity of matrix decomposition and requires additional restrictions or regularizations.

Integrative clustering of multiple genomic data types (iCluster) used likelihood maximization to build a joint latent variable model, with the “weights” matrix shared between data types. L1-norm penalization was used to limit the loadings [63]. Its further development, iCluster+, now allows a variety of different data types, including binary and categorical data. The potential of the algorithm for miRNA:mRNA integration has also been used in the group structured tight iCluster method (GST-iCluster) [64].

Joint and Individual Variation Explained (JIVE) was proposed as an extension of the Principle Component Analysis (PCA) approach. It decomposes the original data into a sum of two informative parts: a low-rank approximation capturing joint structures between data types, and an approximation capturing the distinct structures for each data type. This was shown to outperform CCA. The method was tested on mRNA and miRNA datasets related to glioblastoma of TCGA and, being supplemented with target prediction tools, identified informative clusters of interacting miRNA and mRNA [65].

One of the most advanced matrix factorization methods is the Multi-Omics Factor Analysis (MOFA) [66]. The method was developed for integration of various levels of omics data and for discovering the main sources of variation in multi-omics datasets. Using a Bayesian approach, MOFA infers a set of hidden or pre-defined factors that capture biological and technical variability in the data. The main paradigm is in line with the representation in Fig. 3 (‘components’ are now called ‘factors’). The specificity of the method is that the weight of matrices is considered to be identical and is estimated for all omics data simultaneously. An advantage of the method is also its ability to work with missing data. If some measurements are not available either for mRNA or for miRNA datasets, they will be imputed. MOFA allows estimating factor importance by assessing the proportion of variance explained by each factor in each dataset. Although this method has not been developed for integration of miRNA and mRNA data, it has a lot of potential for this application.

Independent component analysis (ICA) is another powerful method to integrate multi-omics data. The algorithms decomposes original data into signals that are as statistically independent as possible [67]. Despite the method being completely unsupervised, it usually finds more biologically relevant signals in the data then PCA and helps cleaning signals from technical biases [68], [69]. In order to increase reproducibility of the analysis, we recently proposed consensus ICA (consICA) [68] and showed its applicability to integration of miRNA:mRNA data in melanoma. A multidimensional version of the method, tensor ICA (tICA), was shown to outperform CCA, iCluster and JIVE in identifying biological sources of data variation [70].

### 2.7 Hybrid methods

Methods that combine information about miRNA targets with experimental observations have the highest potential for context-dependent integration of miRNA and mRNA data. For example, we presented a basic user friendly tool CoExpress for the analysis of miRNA:mRNA co-expression that used information from the TargetScan database for filtering potentially linked miRNAs and mRNAs [13].

Context-specific interactions between miRNA and mRNA were also investigated in context-specific microRNA analysis (CoSMic) [58]. This algorithm combines sequence-based predictions by TargetScan and other tools with miRNA and mRNA expression data (Spearman correlation) and focuses only on miRNAs that are active in a specific subgroup of samples. The authors demonstrated in a well-controlled cell line experiment that their method efficiently filters out false positive interactions and helps identifying context-specific targets.

Another example is the miRNA master regulator analysis (MMRA), which starts from a differential analysis of miRNA and mRNA expression and then looks for miRNAs whose targets are enriched among differentially expressed mRNAs. After this, a network is built around each miRNA. miRNAs with the highest potential to explain subtype-specific mRNAs are selected (a stepwise linear regression is used to predict mRNA expression by miRNA) [60]. The approach was further developed in clustered miRNA master regulator analysis (ClustMMRA), and tested on epithelial to mesenchymal transition in triple negative breast cancer cells [71]. Now, instead of analysis of each specific miRNA, genomic clusters of miRNAs are considered. Targets of miRNAs were predicted using a combination of tools, including TargetScan and miRTarBase.

A similar paradigm was realized in the anamiR R/Bioconductor package. Experimental data undergo differential expression analysis, correlation and intersection with databases of predicted or validated targets and functional annotation of both miRNA and mRNA data [72]. The authors developed a function-driven analysis workflow to identify miRNA-gene interaction pairs among those participating in significant pathways.

## 3 Summary

Here we provide an overview of the most common and promising approaches that were developed over the past decade to integrate miRNA and mRNA expression data in order to gain a deeper understanding of the gene regulatory fine-tuning events in cellular processes. Historically, a main emphasis has been placed on miRNA-target prediction methods. However, despite recent experimental advances with regards to next generation sequencing analysis of cellular transcriptomes, the main issue of *in silico* target prediction remains the limited accuracy and low agreement between the tools. Even databases with experimentally validated targets and targets based on curated literature mining suffer from little congruence and not much has changed since an initial assessment of precision and sensitivity of available tools was published by Alexiou et al. [73].

**Fig. 4:**
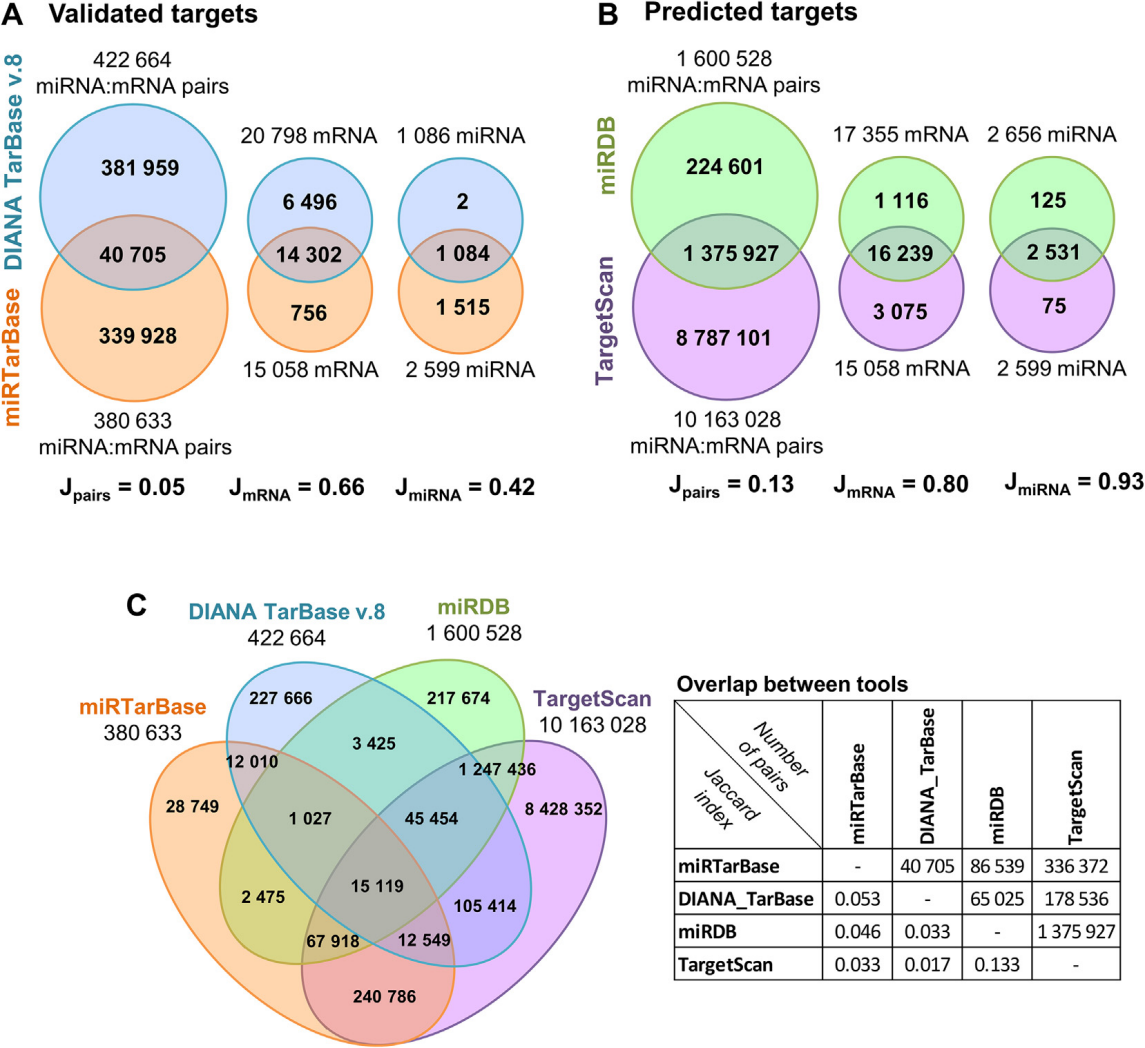
Limited overlap of validated (A) and predicted (B) miRNA targets. For TargetScan, all available miRNA:mRNA pairs including conserved and non-conserved sites and miRNAs were used. To avoid biases due to the selection of miRNAs and mRNAs, we separately intersected lists of miRNAs and mRNAs considered in the tools. The Jaccard index is reported beneath each Venn diagram. (C) Overview of all 4 tools. The number of overlapping miRNA:mRNA pairs as well as the corresponding Jaccard indexes are shown in the table.

To illustrate this point, the intersection between different tools is visualized in Fig. 4. First, the overlap of experimentally validated targets from DIANA TarBase v8 and miRTarBase is shown. Both tools are widely used in the scientific community, but not much attention is given to the discrepancy between these two databases. From our observations for human miRNAs, only ~10% of the recorded miRNA targets are common between these databases (Jaccard index J_pairs_ = 0.05). At the same time, Jaccard indexes for the considered miRNA and mRNA lists are 0.42 and 0.66, respectively, and cannot explain such low overlap of the pairs. Interestingly, prediction algorithms have a slightly higher accordance (Fig. 4B) but this is most probably linked to the extremely high number of reported miRNA:mRNA pairs. Indeed, TargetScan, in its most unrestricted configuration, which includes both conserved and non-conserved sites as well as miRNAs, reported ~26% of all possible miRNA:mRNA pairs as potentially possible. An intersection of all four databases (Fig. 4C), resulted in a somewhat higher concordance (J_pairs_ = 0.046) between miRTarBase and MirDB when predicted and validated targets were combined. The overall very low agreement between different methods and databases can be explained, to some extent, by the notion of context-specific interactions between miRNAs and mRNAs. Indeed, as one miRNA can regulate many mRNAs, the concentration of an active miRNA strongly depends on stoichiometric flux balance with all its interactors. In addition to mRNAs, miRNAs can also bind to lncRNAs or circRNAs that may act as a miRNA sponges [74] or miRNAs may be degraded via target RNA-directed miRNA degradation (TDMD) [75]. Considering the complexity of cellular gene regulatory models, which involve many players with unknown binding affinities and interaction potential, we reckon that a combination of validated experimental data with prediction algorithms trained on more and better high throughout datasets will eventually lead to a more accurate forecast of miRNA targets.

It is a widely accepted simplification that miRNA/mRNA interactions are generally representing a negative correlation, an observation backed by most experimental data. However, several factors can reduce observable negative correlations: (i) co-existence of miRNAs and their targets in specific cell types can lead to a positive correlation in an experiment where several cell types are considered; (ii) as previously shown, miRNA responses to a stimulus may be delayed compared to mRNA responses and thus can only be captures in a time-course experiment [13]; (iii) finally, a scatter plot of miRNA and its targets should have a shape of a triangular area rather than of a simple linear dependency (an absence of miR should not lead to an increase of its target mRNA, if it was not produced beforehand) [55].

Moreover, it is important to consider the number of samples required for the different types of data integration. If methods based on differential expression analysis and target databases require only few samples (enough to find differences between two conditions), correlation-based approaches already need around 10 independent conditions to ensure a bell-shaped correlation distribution under the null hypothesis, i.e. independence of miRNA and mRNA. Semi-supervised matrix factorization methods, such as MOFA, may work when the number of samples is twice higher than the number of estimated factors. Finally, data-driven ICA is sensitive to the number of samples. From our experience, at least 4 samples are needed per one independent component, with 40–50 components required for a good interpretation and associating of the components to the clinical information [67]. Thus, hundreds of samples are needed for such an approach.

Acquisition of high quality datasets of both miRNA and mRNA expression profiles opens the door to apply these advanced deconvolution methods (MOFA, JIVA, ICA) on one side and modern deep-learning models that predict binding affinities on the other side. In future, we may expect to see inclusion of target predictions into matrix factorization algorithms, for example as a regularization factor taken into account during building of the metagene matrices. At the same time, deep learning models can take into account the context of miRNA:mRNA interaction, if enough data are available. Together, these approaches will certainly help to provide improved miRNA targetome predictions in the near future.

## Declaration of Competing Interest

The authors declare that they have no known competing financial interests or personal relationships that could have appeared to influence the work reported in this paper.
